# Mass cytometric analysis of circulating immune landscape in primary central nervous system lymphoma

**DOI:** 10.3389/fimmu.2025.1658015

**Published:** 2025-10-15

**Authors:** Yuchen Wu, Liwei Lv, Jing Liu, Xuefei Sun, Chunji Gao, Shengjun Sun, Nan Ji, Wenjing Wang, Yuanbo Liu

**Affiliations:** ^1^ Department of Hematology, Beijing Tiantan Hospital, Capital Medical University, Beijing, China; ^2^ Department of Hematology, Beijing Tongren Hospital, Capital Medical University, Beijing, China; ^3^ Department of Hematology, Chinese People’s Liberation Army (PLA) General Hospital, Beijing, China; ^4^ Neuroimaging Center, Beijing Tiantan Hospital, Capital Medical University, Beijing, China; ^5^ Department of Neurosurgery, Beijing Tiantan Hospital, Capital Medical University, Beijing, China; ^6^ Beijing Institute of Hepatology, Beijing YouAn Hospital, Capital Medical University, Beijing, China

**Keywords:** primary central nervous system lymphoma (PCNSL), mass cytometry, T cell exhaustion, tumor microenvironment, immunosuppression

## Abstract

**Introduction:**

The peripheral immune profiles of patients with primary central nervous system lymphoma (PCNSL) remain poorly characterized. Investigating immune dysregulation in PCNSL may help elucidate the underlying disease mechanisms.

**Methods:**

We aimed to define the circulating immune landscape in PCNSL by characterizing the immune cell profiles in 16 patients and 6 healthy participants using mass cytometry.

**Results:**

Patients exhibited significant alterations in peripheral blood mononuclear cells, including expansion of CD45RO+ classical monocytes (p=0.017), reduced intermediate subsets (p=0.01), and elevated CD38 expression (p<0.001). The number of terminally differentiated CD8+CD57+ T cells increased (p=0.013), and treatment induced effector T cell (CD8+ T effector/effector memory cells, p<0.05) expansion, accompanied by co-upregulation of CD38, HLA-DR, and CD107a (p<0.01). Patients < 60 years had higher frequencies of CD8+ naïve T cells (p<0.05), and progressive disease correlated with CD56^bright^NK cell accumulation (p<0.01).

**Conclusion:**

the circulating immune landscape in PCNSL is characterized by skewed monocyte activation, T cell terminal exhaustion, and chemotherapy-induced effector T cell expansion. Our findings link peripheral immune features to the tumor microenvironment biology. Understanding these systemic immune alterations may provide insights into tumor immune evasion and offer a roadmap for reversing PCNSL-associated immunosuppression.

## 1 Introduction

Primary central nervous system lymphoma (PCNSL), the diffuse large B cell lymphoma (DLBCL) of the central nervous system (CNS), is a rare but highly aggressive lymphoma confined primarily to the brain, leptomeninges, spinal cord, and intraocular regions. Despite advancements in treatment strategies and marked improvement in patient survival rates, due to its unique environment, the CNS remains a challenging site for both diagnosis and therapy (1).

Although conventionally regarded as an immune-privileged site (2), the CNS engages in complex bidirectional interactions with the immune system (3). This dynamic interplay between the CNS and immune system extends beyond the CNS, potentially shaping the tumor microenvironment (TME) by modulating peripheral immune responses. In PCNSL, the TME is predominantly characterized by infiltration of CD8+ T cells and CD163+ macrophages (4), displaying substantial intra- and intertumoral heterogeneity. CD4+ T cells are primarily localized around the tumor margins, whereas CD/8+ T cells predominate within the tumor core (5). CD8+ T cells in PCNSL selectively express exhaustion markers but do not express LAG-3 or activation markers unlike in systemic DLBCL (1, 6, 7). Furthermore, CD4+ follicular helper T cells and FOXP3+ regulatory T cells are scarce, and CD56+ T cells are nearly absent (5, 7–10). Additional components of the TME, such as macrophages, YKL-40, the PD-1/PD-L1 axis, osteopontin, and HLA loss, are associated with poor prognosis. Thus, the TME drives PCNSL progression and represents a potential therapeutic target (11).

However, the practical utility of these findings remains limited as obtaining PCNSL tissue samples is challenging. Although peripheral blood samples are more accessible than tumor tissues, research into the peripheral immune status of patients with PCNSL is limited.

Hence, this study aimed to define the circulating immune landscape in PCNSL using mass cytometry compared to healthy controls. Mass cytometry, a high-dimensional single-cell technology capable of analyzing over 40 cellular parameters simultaneously, enables a more comprehensive profiling of immune cell populations than conventional flow cytometry by uncovering immune population dynamics and identifying rare cell types more effectively. Thus, investigating circulating immune alterations in PCNSL through mass cytometry could offer novel insights into the diagnosis and prognosis of this highly heterogeneous disease.

## 2 Methods

### 2.1 Patient recruitment, follow‐up, and therapy

This study design was reviewed and approved by the Ethics Committee of Beijing Tiantan Hospital affiliated with Capital Medical University. All patients and healthy participants were recruited from the Department of Hematology, Beijing Tiantan Hospital.

The inclusion criteria for patients see in **Supplementary Material 1**. A total of 16 patients with PCNSL meeting the criteria, and 6 healthy participants were enrolled. Peripheral venous blood samples were collected from the patients in a fasting state before treatment and after three cycles of therapy, and peripheral blood mononuclear cells were isolated for analysis. Peripheral venous blood of the healthy participants was also collected in a fasting state. The treatment regimen details and response evaluation see in **Supplementary Table 2**.

### 2.2 Mass cytometry

Peripheral blood was collected in EDTA tubes and processed within 6 hours using SepMate tubes for PBMC isolation. Cells were stained with cisplatin, counted, and stored at −80 °C. Two antibody panels, one with 34 and the other with 25 antibodies, were used to sort PBMCs from the patients and healthy participants (**Supplementary Tables 3**, **4** presents the details of monoclonal antibodies). PBMCs were surface-stained with lanthanide-labeled antibodies, washed, and stained with intracellular markers, according to the manufacturer’s instructions. After two additional washes, the cells were labeled with a DNA intercalating agent and analyzed using a Helios mass cytometer (Fluidigm, CA, USA) to acquire high-dimensional single-cell data.

### 2.3 Statistics

CyTOF data were exported in.fcs format. After preprocessing, individual live cell data from each sample were manually gated and exported from Cytobank. Arcsine normalization was applied to identify cell clusters in an unbiased manner. The high-dimensional data were subjected to PhenoGraph analysis using the R package ‘cytofkit (v1.10.0)’ with default settings (12). Dimensionality reduction was performed using *t*-distributed stochastic neighbor embedding (t-SNE; perplexity = 45, iterations = 2500, theta = 0.5), and the changes in cluster frequency, heterogeneity, and marker expression between the two groups were visualized. Phenotypically similar clusters were manually merged.

Data were visualized using the R packages ‘pheatmap (v1.0.12)’ and ‘ggplot2 (v3.3.5)’. Marker expression levels and cell counts within each cluster were analyzed using R. The Wilcoxon rank-sum test was used to compare marker expression levels and cell counts between groups. Given the exploratory nature of this high-dimensional analysis and the multiple comparisons performed, we employed a two-tiered approach to significance. Nominal statistical significance was set at a two-sided P value < 0.05. To control for false discovery, P-values were further adjusted using the Benjamini-Hochberg method, with an FDR-corrected q-value < 0.05 considered statistically significant after correction. Rank-sum tests were also employed to compare cell clustering among patients with different clinical features. A two-sided P value <0.05 was considered statistically significant. The defining markers for major immune lineages and their key subsets are summarized in **Table 1**. The specific antibody panels used for deep immunophenotyping are detailed in **Supplementary Tables 5**, **6**.

**Table 1:** Universal markers for defining major immune cell lineages.

Major lineage	Defining markers	Key subsets & defining markers
T Lymphocytes	CD3+	Helper T: CD3+CD4+
		Cytotoxic T: CD3+CD8+
		Naïve: CD45RA+CCR7+
		Memory: CD45RO+
		Terminal Effector/Exhausted: CD57+
		Activated: HLA-DR+, CD38+
B Lymphocytes	CD19+, CD20+	Naïve/Memory: CD27
		Plasmablast/Plasma Cell: CD38++, CD138+
NK Cells	CD3-, CD56+	Cytotoxic (CD56dim): CD16+, Perforin+
		Immunoregulatory (CD56bright): CD16-
Monocytes	CD14+,CD16+, HLA-DR+	Classical: CD14++CD16-
		Intermediate: CD14++CD16+
		Non-classical: CD14+CD16++
Dendritic Cells	CD3-,CD19-,CD14-, CD56-, HLA-DR++	plasmacytoid DC: CD11clow

## 3 Results

### 3.1 Circulating PBMC profile of patients with PCNSL

**Figure 1:**
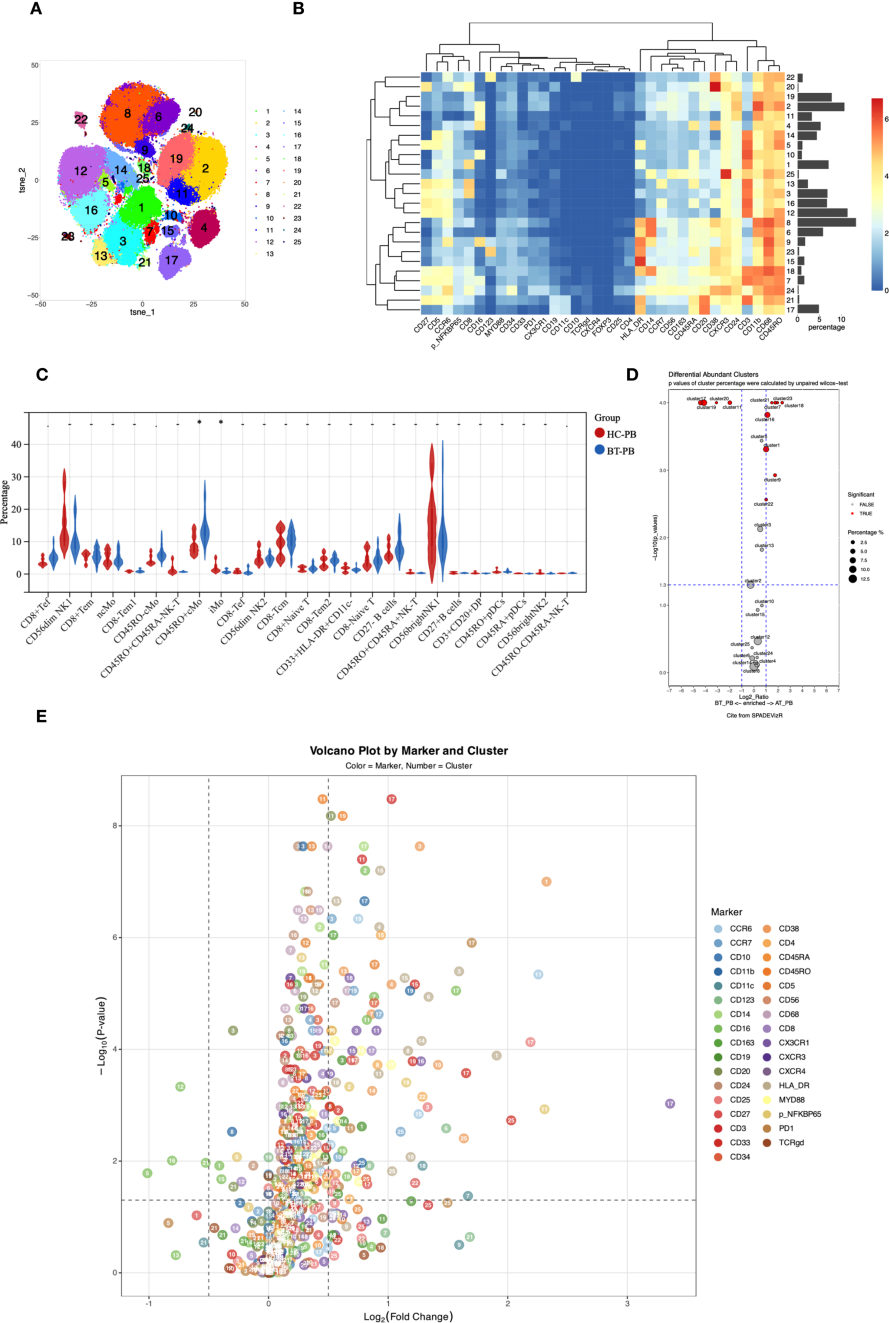
**(A)** *t*-SNE plot showing high-dimensional clustering of 25 immune cell populations identified from PBMCs resulting in the identification of 25 distinct immune cell populations. **(B)** Heatmap showing the relative expression levels of selected markers among the 25 cell subsets identified by t-SNE clustering, as shown in **(A)**. **(C)** Violin plots from nonparametric testing comparing treatment-naive patients with PCNSL and healthy participants. CD45RO+ classical monocytes (Panel1-C08) were significantly higher (p=0.017), whereas the proportion of intermediate monocytes (Panel1-C09) was lower (p=0.01), in untreated patients than in healthy participants. **(D)** Cluster distribution discrepancy between with PCNSL before and after treatment analyzed using the SPADEVizR method. **(E)** Volcano plot showing differential expression of markers in patients with PCNSL before and after treatment.

The clinical characteristics of the patients are summarized in **Table 2**. Twenty-five immune cell populations were identified using Panel 1 (**Table 3**, **Figure 1**), with T cells accounting for the highest proportion (43.47%, 11 subpopulations), followed by monocytes (26.09%, 4 subpopulations) and NK cells (21.95%, 4 subpopulations). B cells (5.07%), plasmacytoid dendritic cells (1.51%), and CD33 HLA-DR myeloid cells (1.53%) were the least dominant cells.

**Table 2:** Clinical characteristics of PCNSL patients.

Characteristics	Patient (N=16)
Age	
Mean±SD	49.00±14.06
Median[min-max]	47.50[30.00,77.00]
Gender	
female	7(43.75%)
male	9(56.25%)
ECOG- PS	
Mean±SD	1.88±1.36
Median[min-max]	1.00[0.0e+0,4.00]
Pathological types	
DLBCL	2(12.50%)
DLBCL-ABC	12(75.00%)
DLBCL-GCB	2(12.50%)
Deep brain involvement	
deep brain involvement	13(81.25%)
no deep brain involvement	3(18.75%)
Elevated serum LDH	
elevated serum LDH	5(31.25%)
normal serum LDH	11(68.75%)
IELSG risk	
high risk	3(18.75%)
intermediate risk	8(50.00%)
low risk	5(31.25%)

**Table 3:** Cluster names of panel1.

Cluster	Name
Panel1-C01	CD8+effectorT
Panel1-C02	CD56dim NK1
Panel1-C03	CD8+Tem
Panel1-C04	ncMo
Panel1-C05	CD8-Tem1
Panel1-C06	CD45RO-cMo
Panel1-C07	CD45RO+CD45RA-NK-T
Panel1-C08	CD45RO+cMo
Panel1-C09	iMo
Panel1-C10	CD8-CD45RA+T
Panel1-C11	CD56dim NK2
Panel1-C12	CD8-Tcm
Panel1-C13	CD8+Naive T
Panel1-C14	CD8-Tem2
Panel1-C15	CD33+HLA-DR+CD11c-
Panel1-C16	CD8-Naive T
Panel1-C17	CD27- B cells
Panel1-C18	CD45RO+CD45RA+NK-T
Panel1-C19	CD56brightNK1
Panel1-C20	CD27+B cells
Panel1-C21	CD3+CD20+DP
Panel1-C22	CD45RO+pDCs
Panel1-C23	CD45RA+pDCs
Panel1-C24	CD56brightNK2
Panel1-C25	CD45RO-CD45RA-NK-T

**Figure 2:**
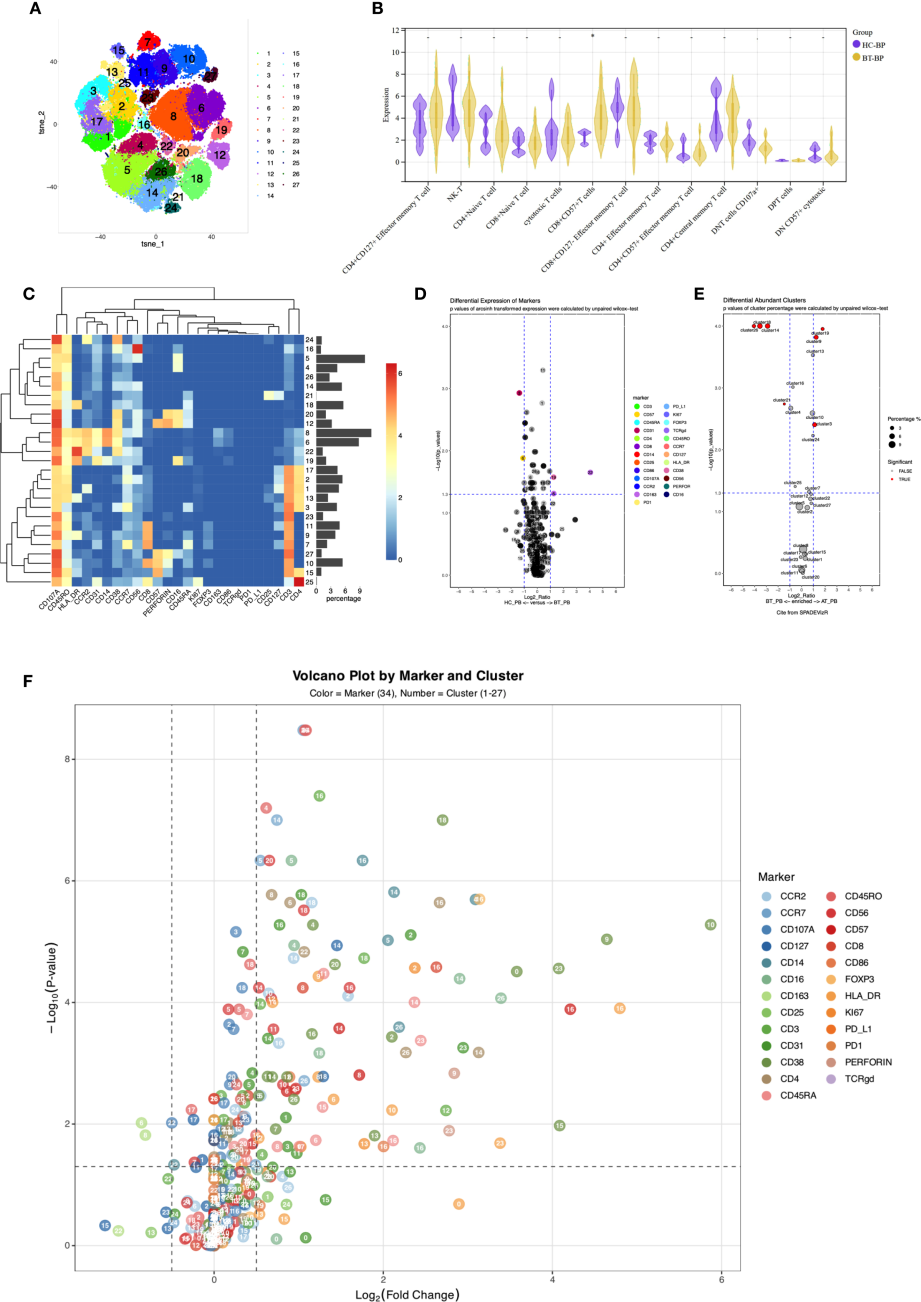
**(A)** *t*-SNE plot illustrating high-dimensional clustering of 27 immune cell populations identified from PBMCs. **(B)** Violin plots from nonparametric testing comparing treatment-naïve PCNSL patients and healthy participants. The frequency of CD8^+^CD57^+^ T cells (Panel2-C10) was significantly increased in PCNSL patients (p = 0.013). **(C)** Heatmap showing relative expression levels of selected markers among the 27 cell subsets identified by the *t*-SNE clustering shown in **(A)**. **(D)** Volcano map highlighting differential marker expression in monocyte and T-cell subsets. Panel2 showed decreased proportion of intermediate monocytes (Panel2-C19; p=0.032) with elevated CD38 expression (p=0.027), decreased CD14 expression in classical monocytes 1 (Panel2-C06; p=0.033), and increased CD163 expression (p=0.049). Panel2-C22 revealed increased expression of CD163 (p=0.022). CD31 was downregulated in CD4+ naive T cells and CD8+CD127- effector memory T cells (Panel2-C03; p=0.0012). **(E)** Cluster distribution discrepancy between patients with PCNSL before and after treatment. **(F)** Volcano plot demonstrating differential expression of markers in patients with PCNSL before and after treatment.

Twenty-seven cell populations were identified using Panel 2 (**Table 4**, **Figure 2**), with T cells divided into 13 subpopulations (41.22%), monocytes into 3 subpopulations (22.16%), and NK cells into 5 subpopulations (20.52%). A plasmacytoid group (1.12%), an innate lymphoid cell group (0.05%), a dendritic cell group (1.33%), and three CD3-negative subpopulations, including two groups of B cells and CD3-HLA-DR+CD45RA+ (13.6%) were also identified.

**Table 4:** Cluster names of panel2.

Cluster	Name
Panel2-C01	CD4+CD127+ Effector memory T cells
Panel2-C02	NK-T cells
Panel2-C03	CD4+Naïve T cells
Panel2-C04	NK1 cells
Panel2-C05	NK2 cells
Panel2-C06	classical Monocytes1
Panel2-C07	CD8+Naïve T cells
Panel2-C08	classical Monocytes2
Panel2-C09	cytotoxic T cells
Panel2-C10	CD8+CD57+T cells
Panel2-C11	CD8+CD127- Effector memory T cells
Panel2-C12	NK3 cells
Panel2-C13	CD4+ Effector memory T cells
Panel2-C14	B cells1
Panel2-C15	CD4+CD57+ Effector memory T cells
Panel2-C16	weakly toxicity NK cells
Panel2-C17	CD4+Central memory T cells
Panel2-C18	CD3-HLA-DR+CD45RA+ cells
Panel2-C19	intermediate monocytes
Panel2-C20	NK4 cells
Panel2-C21	innate Lymphoid Cells
Panel2-C22	dendritic cells
Panel2-C23	CD107a+DNT cells
Panel2-C24	plasma cells
Panel2-C25	DPT cells
Panel2-C26	Bcells2
Panel2-C27	DN CD57+ cytotoxic

### 3.2 Immune subset differences between patients with PCNSL and healthy participants

#### 3.2.1 Monocyte dysregulation in patients with PCNSL

In the peripheral blood of patients with PCNSL before treatment (PCNSL-BT), Compared to healthy participants, untreated PCNSL patients showed a higher proportion of CD45RO+ classical monocytes (Panel1-C08; p=0.017) and a lower proportion of intermediate monocytes (Panel1-C09; p=0.01), although these findings were not significant after adjustment for multiple comparisons. CD38 (p=0.00019) and CD68 (p=0.049) were upregulated in the intermediate monocytes of patients, with the CD38 upreduction in intermediate monocytes remaining significant after FDR correction (q < 0.05).

CD14 expression in two classical monocyte clusters (Panel1-C06/C08) was lower in patients (p=0.027 and 0.013, respectively), and CD11c expression was reduced in Panel1-C08 (p=0.019). Furthermore, CCR7 expression in non-classical monocytes (Panel1-C04) was significantly reduced (p=0.0045) in the patients.

Verification using Panel 2 confirmed a reduced proportion of intermediate monocytes (Panel2-C19) in patients (p=0.032), accompanied by elevated CD38 expression (p=0.027). In classical monocytes 1 (Panel2-C06), CD14 was downregulated (p=0.033) and CD163 was upregulated (p=0.049), further supporting monocyte subset dysregulation in PCNSL.

#### 3.2.2 Enhanced survival signals but limited effector function in T cells of patients with PCNSL

Analysis of T cell subpopulations using panel 2 revealed an increased proportion of CD8+CD57+T cells (Panel2-C10) in the PCNSL-BT group (p=0.013), alongside decreased CCR2 expression (p=0.017), indicating an accumulation of terminally differentiated T cells with diminished migratory capacity.

CD127 was significantly upregulated in CD4+CD127+ effector memory T cells and CD8+CD127- effector memory T cells (Panel2-C01 and C11, respectively; p=0.0017 and 0.00051, FDR-corrected q < 0.05), strongly suggesting enhanced survival signaling in these subsets. A similar upregulation trend was also observed in CD4+ central memory T cells (Panel2-C17; p=0.033). CD4+ central memory T cells (Panel2-C17) also exhibited elevated CCR2 and CCR7 expression, potentially facilitating tumor-directed migration.

CD31 was downregulated in CD4+ naive T cells and CD8+CD127- effector memory T cells (Panel2-C03 and C11, respectively; p=0.0012 with q<0.05 and 0.043, respectively), possibly impairing transendothelial migration. Furthermore, reduced CD107a expression in CD8+CD127- effector memory T cells and CD4-CD8- double-negative T cells (Panel2-C11 and C23, respectively; p=0.01 and 0.017, respectively) indicated impaired degranulation function. Downregulated CD38 in CD4+ naive T cells and CD8+ naive T cells (Panel2-C03 and C07, respectively; p=0.0024 with q<0.05 and 0.033, respectively) suggested insufficient activation or enhanced inhibitory regulation, potentially delaying immune response initiation. Reduced CCR7 expression in CD56^dim^NK1 cells (Panel1-C02; p=0.033) suggested impaired NK cell migration to lymphoid tissues and weakened immune surveillance.

Analysis using Panel 1 revealed significantly increased expression of CD16 (p=0.0008 with q<0.05), CD45RO (p=0.021), and CXCR3 (p=0.049) in CD8-CD45RA+ T cells (Panel1-C10), with elevated CD16 expression, indicating potentially enhanced ADCC (Antibody-Dependent Cell-Mediated Cytotoxicity) function, and significantly increased CXCR3 expression, suggesting a migration tendency toward CXCL9/10/11-expressing tissues. Downregulated CD24 in NK-T cells (Panel1-C07; p=0.021) may affect their interaction with ligands and transmit inhibitory signals. Elevated CCR7 expression in CD8- naive T cells (Panel1-C16) compared to that in normal controls (p=0.017) suggested enhanced trafficking to secondary lymphoid organs and peripheral immune activation, whereas lower expression of CD5, CCR6, and CD27 compared to those in normal controls (p=0.021, 0.021, and 0.049, respectively) implied impaired differentiation potential of naive T cells.

Overall, T cell subsets in patients with PCNSL exhibited marked CD4/CD8 functional differentiation. CD4+ central memory T cells exhibited activation phenotypes, including upregulation of CD127 and CCR7, supporting anti-tumor responses by sustaining immune memory and survival signals. In contrast, CD8+ T cells displayed typical exhaustion phenotypes characterized by functional inhibition (downregulation of CD107a and CD31) and terminal differentiation (upregulation of CD57 and downregulation of CCR2), suggesting suppression of effector activity within the TME. This functional imbalance between CD4+ and CD8+ T cells may collaboratively contribute to tumor immune evasion.

### 3.3 Global immune activation in patients after treatment

#### 3.3.1 Anti-tumor immune responses driven by treatment-induced expansion of effector lymphocytes and attenuation of regulatory subsets

After treatment, we observed significant increases in the proportions of several immune cell subsets, including CD8+ effector T cells (Panel1-C01), CD45RO+CD45RA- NK-T cells (Panel1-C07 and C18), intermediate monocytes (Panel1-C09), CD8- naive T cells (Panel1-C16), CD3+CD20+ double-positive T cells (Panel1-C21), CD45RO+ pDCs (Panel1-C22), and CD45RA+ pDCs (Panel1-C23) (p<0.05, q<0.05). Conversely, significant decreases were noted in the proportions of CD27- B cells (Panel1-C17), CD27+ B cells (Panel1-C20), CD56^dim^NK2 cells (Panel1-C11), and CD56^bright^NK1 cells (Panel1-C19)(p<0.05, q<0.05).

Panel2 corroborated these findings, showing widespread activation of cytotoxic functions post-treatment with significant increases in proportions of CD4+ naive T cells (Panel2-C03), cytotoxic T cells (Panel2-C09), and intermediate monocytes (Panel2-C19). In contrast, the proportions of innate lymphoid cells (Panel2-C21), two B cell subsets (Panel2-C26 and Panel2-C14), and CD3-HLA-DR+CD45RA+ cells (Panel2-C18) decreased, these reductions remained significant after FDR correction.

#### 3.3.2 Functional activation of T/NK cells indicated by post-treatment changes in CD38 and HLA-DR expression

Following treatment, CD38 and HLA-DR were significantly upregulated in T cell and NK cell subpopulations. This was evident in CD8+ effector T cells (Panel1-C01) and CD8+ effector memory T cells (Panel1-C03) in Panel 1 and in CD8+ naive T cells (Panel2-C07) in Panel 2, indicating enhanced metabolic activation and co-stimulatory signaling. Furthermore, CD25 was significantly upregulated in CD8+ effector memory T cells (Panel1-C03) after FDR-corrected(q < 0.05).

Elevated expression of CX3CR1 (chemokine receptor for CX3CL1) in CD8- naive T cells (Panel1-C16) might promote migration to the CNS. Significant upregulation of CD38 and HLA-DR was also observed in CD8-CD45RA+ T cells (Panel1-C10), mirroring changes in CD8+ effector cells. Verification using Panel2 confirmed increased CD38 and HLA-DR expression in CD4+ naive T cells (Panel2-C03), indicating transition from a resting to an activated phenotype.

Significantly increased expression of CD38, HLA-DR, and CCR7 in CD45RO-CD45RA- NK-T cells (Panel1-C25) suggested activation and altered migration or homing potential. These findings were verified using Panel 2, revealing significant upregulation of CD38 and HLA-DR in NK-T (Panel2-C02), cytotoxic T (Panel2-C09), CD8+CD57+ terminally differentiated T (Panel2-C10), and CD107A+ double-negative T cells (Panel2-C23). Notably, upregulated CD107A indicated widespread activation of cytotoxic function.

Additional NK cell alterations included upregulation of NF-kB and CCR6 in CD56^dim^NK2 cells (Panel1-C11) and NF-kB, CD33, and CD11b in CD56^bright^NK1 cells (Panel1-C19). CD27 expression increased significantly in CD45RO+ pDCs (Panel1-C22), (all q < 0.05), as determined by Wilcoxon testing with FDR correction.

In other subpopulations, upregulation of HLA-DR in non-classical monocytes (Panel1-C04) and CD45RO+ classical monocytes (Panel1-C08) and upregulation of HLA-DR and CD123 in CD45RO- classical monocytes (Panel1-C06) indicated strengthened antigen-presenting capabilities. Increased HLA-DR, CCR6, and CD33 expression was observed in CD33+HLA-DR+CD11c- myeloid cells (Panel1-C15). The significantly increased expression of HLA-DR (p=0.049 and 0.031, respectively), CD86 (p=0.015 and 0.022, respectively), and CD163 (p=0.007 and 0.013, respectively) in classical monocytes of Panel 2 (Panel2-C06 and C12, respectively) indicated the existence of pro-inflammatory phenotypes in an immunosuppressive microenvironment(p < 0.05 for all; FDR-corrected q < 0.05).

### 3.4 Correlation between immune profiling and clinical characteristics in PCNSL

All patients received high-dose methotrexate-based induction chemotherapy. Twelve patients (75.0%) were treated with the R-MAD regimen, while 4 patients (25.0%) received orelabrutinib combined with R-MAD (Ore-R-MAD) therapy (treatment details are provided in the **Supplementary Table 2**). After completing 3 cycles of induction treatment, response evaluation showed an overall response rate (ORR, including CR+PR) of 68.8% (11/16). Among these, 7 patients (43.7%) achieved complete response (CR), and 3 patients (18.8%) achieved partial response (PR). Five patients (37.5%) experienced disease progression (PD) during this period. With a median follow-up of 34 months for censored patients, the median progression-free survival (PFS) was 4.5 months (95% confidence interval [CI], 2.8 to 9.0 months). The median overall survival (OS) was not reached. (**Supplementary Figure 1**).

Among the 16 patients, those aged < 60 years exhibited a higher proportion of CD8+ naive T cells (Panel1-C13) than those aged ≥ 60 years. Patients with an Eastern Cooperative Oncology Group (ECOG) score ≤1 had higher proportions of CD8+ effector T cells (Panel1-C01) than those with ECOG >1 (p<0.05). After three cycles of treatment, patients with progressive disease had significantly higher proportions of CD33+HLA-DR+CD11c- cells (Panel1-C15), CD56^bright^NK1 cells (Panel1-C19), and CD4+CD8+ double-positive cells (Panel2-C25) in peripheral blood than patients who achieved complete or partial response (p<0.01).

## 4 Discussion

Our study leveraged CyTOF data to comprehensively characterize the peripheral immune landscape in PCNSL. We found that patients with PCNSL exhibited distinct alterations in monocyte and T cell subsets compared to healthy participants. These findings fill a critical gap in understanding how systemic immune dysregulation contributes to CNS tumor immunity, as prior studies have focused primarily on tumor-intrinsic or TME-specific mechanisms (13, 14).

In the PCNSL TME, T cells and monocytes/macrophages are the predominant immune cell populations. PCNSL typically displays a perivascular growth pattern, with 30% of patients showing interstitial distribution and perivascular reactive T cell infiltration (9). CD4+ T cells localize around tumor borders, whereas CD8+ T cells infiltrate tumor cores and frequently express the exhaustion marker TIM-3 (10). The infiltration level of effector T cells in PCNSL is lower than in peripheral DLBCL (15), due to the loss of HLA I/II expression, activation of PD-1/PD-L1 axis, and immunosuppressive factors (such as interleukin-10 and tumor growth factor-β) (16–21). PD-1/PD-L1 blockade has emerged as a major immunotherapeutic strategy across multiple cancers, yet its efficacy is often limited by intrinsic and acquired resistance mechanisms related to antigen presentation defects and immunosuppressive microenvironments (22). Although the brain was considered immune-privileged, recent studies show that brain tumors increase blood–brain barrier permeability, revealing shared features between the CNS and peripheral TMEs (23).

T cell exhaustion and impaired migration characterize the dysfunctional immune state in PCNSL. We observed that the proportion of CD8+CD57+ T cells in the peripheral blood of patients with PCNSL increased significantly. The upregulation of CD57, a marker of terminally differentiated T cells, may reflect T cell exhaustion caused by persistent antigen stimulation (24, 25). This observation is consistent with their exhausted phenotype in the TME (7). The downregulation of CCR2 in CD8+CD57+ T cells may affect their migration to TME enriched with chemokines (such as CCL2), thus weakening the local anti-tumor response. Although CD8+ effector T cells show enhanced memory-associated markers (e.g., upregulation of CD45RO), similar to prior observations of an effector memory phenotype (14) and upregulation of Th1 related chemokine receptor (CXCR3) in PCNSL infiltrating CD4+ and CD8+ T cells. This suggests the potential of the tumor to differentiate into cytotoxic or inflammatory phenotypes. However, the degranulation ability (indicated by downregulation of CD107a) (26) and migration function (indicated by downregulation of CD31) of these cells were impaired, indicating a contradictory state of activation and dysfunction.

The downregulation of CD38 in CD8+ naive T cells may reflect the lack of antigen recognition or costimulatory signals, resulting in the inability of naive T cells to effectively initiate differentiation. The impaired metabolism of NAD+ may affect the energy supply of cells, hinder their differentiation into functional effector T cells, and indirectly lead to the weakening of the overall anti-tumor immune response (27). This paradoxical immune activation coexisting with dysfunction mirrors the immunosuppressive TME dynamics, where PD-1/PD-L1 axis activation and HLA loss further dampen effector responses. The downregulation of CD38 and CD107a in T cells might improve after treatment; however, the upregulation of CD57 was not reversed.

Monocyte remodeling further reflects TME-driven immunologic reprogramming in PCNSL. Infiltrating tumor-associated macrophages (TAMs) are derived from the peripheral circulating monocyte macrophage cell line (5). Similar to that observed for T cells, TAM infiltration was less in PCNSL than in DLBCL (28). The proportion of intermediate monocytes (CD14++CD16+) decreased, whereas the activation markers, CD38 and CD68, were upregulated, suggesting local pro-inflammatory signal activation. This decrease in intermediate monocytes may be associated with the migration of these cells to the TME and differentiation into TAMs. We observed that CD163 was upregulated on classical monocytes, suggesting that peripheral monocytes may have shifted toward the M2 phenotype. In addition, the significant upregulation of CD38 in intermediate monocytes may promote immunosuppression in the TME (29). CCR7 was downregulated in non-classical monocytes (CD14+CD16++), suggesting limited migration ability to immune surveillance sites. In peripheral DLBCL, the proportion of classical and intermediate monocytes in peripheral blood increased and exhibited an inflammatory phenotype, whereas non classical monocytes decreased (9, 30). The significant increase of CD45RO+ classical monocytes and the upregulation of CD163 in the peripheral blood of patients with PCNSL may indicate that the TME polarizes classical monocytes through inflammation-related pathways. This imparts pro-inflammatory properties and is consistent with the phenomenon of inflammatory phenotype induction in the classical and intermediate monocytes in DLBCL. However, the function of monocytes in TME remains unclear and requires further analysis in combination with tissue positioning.

Peripheral immune profiles correlate with clinical outcomes, offering prognostic insights. In this study, the proportion of CD8+ naive T cells in the peripheral and bone marrow of patients aged less than 60 years was significantly higher than that of patients aged ≥ 60 years. This suggests that young patients have a stronger thymic output (27), which is consistent with the improved prognosis of young patients with PCNSL. However, whether CD8+ naive T cells have the characteristics of recent thymic emigrants, such as epigenetic stability and interleukin signal sensitivity, and whether increased thymic output translates into a higher proportion of tumor infiltrating effector T cells, remains to be verified. The proportion of peripheral CD8+ effector T cells in patients with PS score ≤ 1 was significantly increased, suggesting that good physical fitness is associated with systemic anti-tumor immune activation.

While this study provides foundational insights into the peripheral immune landscape of PCNSL, future investigations could further strengthen its translational impact. The current sample size, though informative for hypothesis generation, warrants expansion in larger cohorts to enhance statistical rigor and account for clinical heterogeneity. Age-sensitive immune parameters, such as naïve T-cell frequencies, may benefit from age-stratified analyses in follow-up studies. Additionally, integrating matched tumor tissue or cerebrospinal fluid profiling with peripheral immune data could clarify mechanistic links between systemic changes and intratumoral biology.

This study must be interpreted considering its limitations. The sample size, though adequate for initial exploration, restricts statistical power for subgroup analyses and elevates type I/II error risk. Additionally, the high-dimensional mass cytometry data required multiple comparisons, and while FDR-adjusted results are provided, several immune phenotypes—including the proposed terminal exhaustion of CD57+CD8+ T cells based on marker expression—await functional validation. Finally, inferences linking peripheral immune changes to the CNS TME remain indirect without paired tissue or CSF correlation. Thus, our findings highlight compelling yet preliminary patterns necessitating confirmation in larger cohorts with functional and anatomical validation.

In conclusion, systemic immune dysregulation in PCNSL is intricately linked to tumor-associated inflammation and immune evasion as evidenced by peripheral T cell exhaustion and M2-polarized myeloid remodeling that mirrored immunosuppressive TME dynamics. Future work integrating functional validation of these subpopulations can facilitate our understanding of PCNSL immune mechanisms and guide the development of potential diagnostic or therapeutic targets.

## Data Availability

The data presented in the study are deposited in the National Genomics Data Center repository, accession number: OMIX012079.
