# Correction: Mass cytometric analysis of circulating immune landscape in primary central nervous system lymphoma

**DOI:** 10.3389/fimmu.2025.1725923

**Published:** 2025-10-21

**Authors:** Yuchen Wu, Liwei Lv, Jing Liu, Xuefei Sun, Chunji Gao, Shengjun Sun, Nan Ji, Wenjing Wang, Yuanbo Liu

**Affiliations:** ^1^ Department of Hematology, Beijing Tiantan Hospital, Capital Medical University, Beijing, China; ^2^ Department of Hematology, Beijing Tongren Hospital, Capital Medical University, Beijing, China; ^3^ Department of Hematology, Chinese People’s Liberation Army (PLA) General Hospital, Beijing, China; ^4^ Neuroimaging Center, Beijing Tiantan Hospital, Capital Medical University, Beijing, China; ^5^ Department of Neurosurgery, Beijing Tiantan Hospital, Capital Medical University, Beijing, China; ^6^ Beijing Institute of Hepatology, Beijing YouAn Hospital, Capital Medical University, Beijing, China

**Keywords:** primary central nervous system lymphoma (PCNSL), mass cytometry, T cell exhaustion, tumor microenvironment, immunosuppression

In the published article, there was a mistake in the author affiliation. Affiliation “Department of Hematology, Beijing Tiantan Hospital, Capital Medical University, Beijing, China” was erroneously given as “Department of Hematology, Beijing Tongren Hospital, Capital Medical University, Beijing, China” for authors Yuchen Wu, Jing Liu, Xuefei Sun, and Yuanbo Liu.

The original version of this article has been updated.

