# Supplementary material for: Mass cytometric analysis of circulating immune landscape in primary central nervous system lymphoma
Source: Front Immunol. 2025 Oct 15;16:1658015. doi: 10.3389/fimmu.2025.1658015 (PMC12584880; doi:10.3389/fimmu.2025.1658015)
Supplement: Supplementary file 1 [file Supplementaryfile1.docx]

Supplementary Material 1 :Inclusion criteria

1. Newly diagnosed with pathologically confirmed DLBCL of the CNS. 2. Diagnosis of peripheral lymphoma was excluded after whole-body PET-CT. 3. No prior treatment. 4. No severe liver, kidney, or heart dysfunction and eligibility for systemic chemotherapy.

Supplementary Table 2: The treatment regimen details and response in PCNSL patients.

| No of  patients | Age | Gender | ECOG- PS | Pathological types | IELSG risk | Induction treatment | Efficacy after 3 cycles |
| --- | --- | --- | --- | --- | --- | --- | --- |
| patient1 | 54 | male | 2 | DLBCL-ABC | intermediate | R-MAD* | PR |
| patient2 | 41 | female | 1 | DLBCL-ABC | intermediate | Ore-R-MAD** | CR |
| patient3 | 59 | female | 4 | DLBCL-ABC | intermediate | R-MAD* | PD |
| patient4 | 36 | male | 0 | DLBCL-ABC | low | R-MAD* | PR |
| patient5 | 40 | male | 1 | DLBCL-ABC | low | R-MAD* | CR |
| patient6 | 30 | male | 2 | DLBCL-ABC | intermediate | R-MAD* | PR |
| patient7 | 63 | female | 1 | DLBCL-ABC | high | R-MAD* | PD |
| patient8 | 39 | female | 1 | DLBCL-ABC | low | R-MAD* | PD |
| patient9 | 54 | male | 1 | DLBCL-ABC | intermediate | R-MAD* | CR |
| patient10 | 34 | male | 4 | DLBCL-ABC | intermediate | Ore-R-MAD** | CR |
| patient11 | 32 | female | 1 | DLBCL-ABC | low | R-MAD* | PD |
| patient12 | 65 | female | 1 | DLBCL-GCB | high | Ore-R-MAD** | CR |
| patient13 | 77 | male | 4 | DLBCL-ABC | high | R-MAD* | PD |
| patient14 | 41 | male | 4 | DLBCL | intermediate | Ore-R-MAD** | PD |
| patient15 | 61 | male | 1 | DLBCL | low | R-MAD* | CR |
| patient16 | 58 | female | 2 | DLBCL-GCB | intermediate | R-MAD* | CR |

*R-MAD

Day 0: Rituximab 375mg/m^2^ IV

Day 1: Methotrexate 3.5g/m^2^ IV within 3.5 hours.

Day 2: Leucovorin rescue every 6 hours until the methotrexate < 0.10 μmol/L.

Day 2: Cytarabine 1-2g/m^2^ IV

Day 1-3: Dexamethasone 10mg IV

**Ore-R-MAD

Oral Orelabrutinib 150mg once a day

Day 0: Rituximab 375mg/m^2^ IV

Day 1: Methotrexate 3.5g/m^2^ IV within 3.5 hours.

Day 2: Leucovorin rescue every 6 hours until the methotrexate < 0.10 μmol/L.

Day 2: Cytarabine 1-2g/m^2^ IV

Day 1-3: Dexamethasone 10mg IV.

Supplementary Table 3: Markers in Panel1

| Marker | Label |
| --- | --- |
| CXCR3 | 141Pr |
| MYD88 | 143Nd |
| CD56 | 144Nd |
| CD14 | 145Nd |
| CD45RA | 146Nd |
| CD5 | 147Sm |
| CD19 | 148Sm |
| CD34 | 149Sm |
| CD10 | 150Sm |
| CD123 | 151Eu |
| CD24 | 152Gd |
| CD163 | 153Eu |
| CD25 | 154Gd |
| CCR6 | 155Gd |
| CD3 | 156Gd |
| CD33 | 158Gd |
| PD1 | 159Tb |
| CD68 | 160Dy |
| CD20 | 161Dy |
| CD11c | 162Dy |
| TCRgd | 163Dy |
| CD45RO | 164Dy |
| CXCR4 | 165Ho |
| p_NFKBP65 | 166Er |
| CD38 | 167Er |
| CD8 | 168Er |
| FOXP3 | 169Tm |
| CD27 | 170Yb |
| CX3CR1 | 171Yb |
| CD11b | 172Yb |
| CCR7 | 173Yb |
| HLA_DR | 174Yb |
| CD4 | 175Lu |
| CD16 | 209Bi |

Supplementary Table 4 Markers in Panel2

| Marker | Label |
| --- | --- |
| CD45 | 112Cd |
| CD3 | 141Pr |
| CD57 | 142Ce |
| CD45RA | 143Nd |
| CD31 | 144Sm |
| CD4 | 145Nd |
| CD8 | 146Nd |
| CD14 | 148Sm |
| CD25 | 149Sm |
| CD86 | 150Sm |
| CD107A | 151Eu |
| CCR2 | 153Eu |
| CD163 | 154Gd |
| PD1 | 155Gd |
| PD_L1 | 156Gd |
| KI67 | 161Dy |
| FOXP3 | 162Dy |
| TCRgd | 163Dy |
| CD45RO | 166Er |
| CCR7 | 167Er |
| CD127 | 168Er |
| HLA_DR | 170Er |
| CD38 | 172Yb |
| CD56 | 173Yb |
| PERFORIN | 175Lu |
| CD16 | 209Bi |

Supplementary Table 5 Annotated Immune Cell Clusters in Panel 1

| Cluster | Name | Marker |
| --- | --- | --- |
| Panel1-C01 | CD8+effectorT | CD3+CD8+CD45RA+CD45RO-CCR7- |
| Panel1-C02 | CD56dim NK1 | CD3-CD56+CD16+PD-1+ |
| Panel1-C03 | CD8+Tem | CD3+CD8+CD45RA-CD45RO+CCR7- |
| Panel1-C04 | ncMo | CD3-CD14-CD16+CX3CR1-CD56-HLA-DR- |
| Panel1-C05 | CD8-Tem1 | CD3+CD8-CD45RA-CD45RO+CCR7- |
| Panel1-C06 | CD45RO-cMo | CD3-CD14+CD16-CX3CR1-HLA-DR+ |
| Panel1-C07 | CD45RO+CD45RA-NK-T | CD3+CD8+CD56+CD45RA-CD45RO+CCR7+ |
| Panel1-C08 | CD45RO+cMo | CD3-CD14+CD16-CX3CR1-HLA-DR+ |
| Panel1-C09 | iMo | CD3-CD14+CD16+CR3CR1lowHLA-DR+ |
| Panel1-C10 | CD8-CD45RA+T | CD3+CD8-CD45RA+CD45RO-CCR7- |
| Panel1-C11 | CD56dim NK2 | CD3-CD56+CD16+PD-1- |
| Panel1-C12 | CD8-Tcm | CD3+CD8-CD45RA+CD45RO+CCR7- |
| Panel1-C13 | CD8+Naive T | CD3+CD8+CD45RA+CD45RO-CCR7+ |
| Panel1-C14 | CD8-Tem2 | CD3+CD8-CD45RA-CD45RO+CCR7- |
| Panel1-C15 | CD33+HLA-DR+CD11c- | CD33+HLA-DR+CD11b-CD11c- |
| Panel1-C16 | CD8-Naive T | CD3+CD8-CD45RA+CD45RO-CCR7+ |
| Panel1-C17 | CD27- B cells | CD3-CD19+CD20+CD27+CD38- |
| Panel1-C18 | CD45RO+CD45RA+NK-T | CD3+CD8+CD56+CD45RA+CD45RO+ |
| Panel1-C19 | CD56brightNK1 | CD3-CD56+CD16-CCR6- |
| Panel1-C20 | CD27+B cells | CD3-CD19+CD20+CD27+CD38+ |
| Panel1-C21 | CD3+CD20+DP | CD3+CD20+DP |
| Panel1-C22 | CD45RO+pDCs | CD33-HLA-DR-CD11b-CD11c- |
| Panel1-C23 | CD45RA+pDCs | CD33-HLA-DR+CD11b-CD11c+ |
| Panel1-C24 | CD56brightNK2 | CD3-CD56+CD16-CCR6+ |
| Panel1-C25 | CD45RO-CD45RA-NK-T | CD3+CD8+CD56+CD45RA-CD45RO- |

Supplementary Table 6 Annotated Immune Cell Clusters in Panel 2

| Cluster | Name | Marker |
| --- | --- | --- |
| Panel2-C01 | CD4+CD127+ Effector memory T cells | CD3+CD4+CD8-CD45RA-CD45RO+CD127+ |
| Panel2-C02 | NK-T cells | CD3+CD4+CD8-CD56+CD45RA-CD45RO+CCR7+ |
| Panel2-C03 | CD4+Naïve T cells | CD3+CD4+CD8-CD45RA+CD45RO-CCR7+ |
| Panel2-C04 | NK1 cells | CD3-CD16+ |
| Panel2-C05 | NK2 cells | CD3-CD16+ |
| Panel2-C06 | classical Monocytes | CD3-CD14+CD16-HLA-DR+CCR2+CD38+CD31+CD107A+ |
| Panel2-C07 | CD8+Naïve T cells | CD3+CD4-CD8+CD45RA+CD45RO-CCR7+ |
| Panel2-C08 | classical Monocytes | CD3-CD14+CD16-CD56+HLA-DR+CCR2+CD38+CD31+CD107A+ |
| Panel2-C09 | cytotoxic T cells | CD3+CD4-CD8+CD107A+CD45RA-CD45RO-CCR7-PERFORIN- |
| Panel2-C10 | CD8+CD57+T cells | CD3+CD4-CD8+CD107A+PERFORIN+CD57+CD45RA-CD45RO+ |
| Panel2-C11 | CD8+CD127- Effector memory T cells | CD3+CD4-CD8+CD45RA-CD45RO+ |
| Panel2-C12 | NK3 cells | CD3-CD56+CD16+CD38+PERFORIN+CD57+CD107A+ |
| Panel2-C13 | CD4+ Effector memory T cells | CD3+CD4+CD8-CD45RA-CD45RO+ |
| Panel2-C14 | B cells1 | CD3-HLA-DR-CD45RO+ |
| Panel2-C15 | CD4+CD57+ Effector memory T cells | CD3+CD4+CD8-CD107A+CD57+CD45RA-CD45RO+PD-1- |
| Panel2-C16 | weakly toxicity NK cells | CD3-CD56+ |
| Panel2-C17 | CD4+Central memory T cells | CD3+CD4+CD8-CD45RA-CD45RO+CCR7+ |
| Panel2-C18 | CD3-HLA-DR+CD45RA+ cells | CD3-HLA-DR+CD45RA+ |
| Panel2-C19 | intermediate monocytes | CD3-CD16+CD14+HLA-DR+CD31+CD107A+ |
| Panel2-C20 | NK4 cells | CD3-CD56+CD16+CD14-CD38+PERFORIN+CD107A+ |
| Panel2-C21 | innate Lymphoid Cells | CD3-CD25+CD127+ |
| Panel2-C22 | dendritic cells | CD3-HLA-DR+CCR2+CD38+ |
| Panel2-C23 | CD107a+DNT cells | CD3+CD4-CD8-CD107A+CD45RA-CD45RO- |
| Panel2-C24 | plasma cells | CD3-CD38+CD107A+ |
| Panel2-C25 | Name | CD3+CD4+CD8+CD45RA-CD45RO+ |
| Panel2-C26 | CD4+CD127+ Effector memory T cells | CD3-CD4+CD127+ |
| Panel2-C27 | NK-T cells | CD3+CD4-CD8-CD107A+PERFORIN+CD57+CD45RA-CD45RO- |


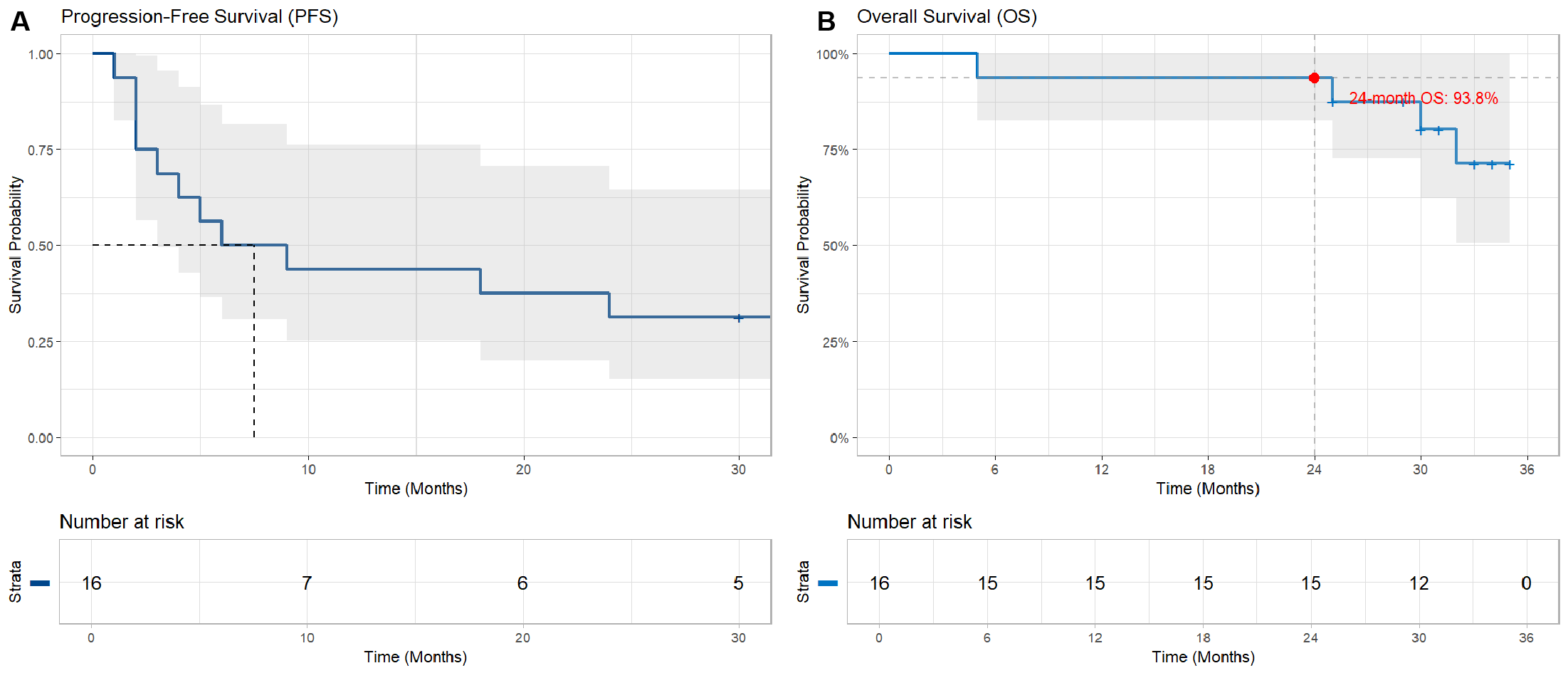


Supplementary Figure 1

A.With a median follow-up of 34 months, the median progression-free survival (PFS) was 4.5 months (95% confidence interval [CI], 2.8 to 9.0 months).

B.The median overall survival (OS) was not reached. The 12- and 24-month OS rates were 93.8% (95% CI, 63.2 to 99.1) and 83.9% (95% CI, 48.3 to 95.9), respectively.
